# Supplementary material for: Neuronal models of TDP-43 proteinopathy display reduced axonal translation, increased oxidative stress, and defective exocytosis
Source: Front Cell Neurosci. 2023 Nov 13;17:1253543. doi: 10.3389/fncel.2023.1253543 (PMC10679756; doi:10.3389/fncel.2023.1253543)
Supplement: Supplementary file 1 [file Data_Sheet_1.docx]

SUPPLEMENTAL FIGURES

**
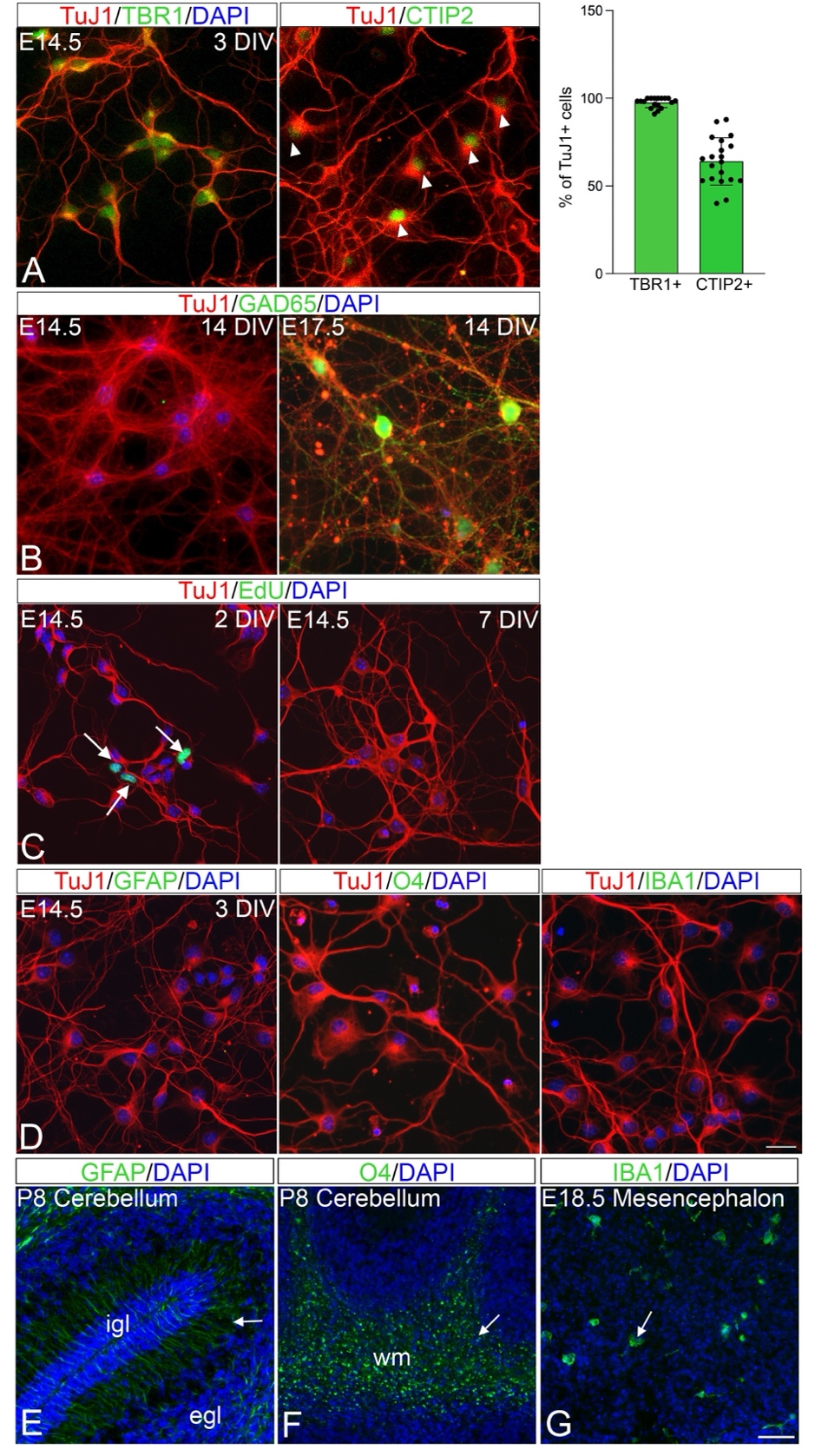
**

**Supplemental figure 1.** **E14.5 primary cortical cultures are strongly enriched in deep-layer glutamatergic neurons.**

(A) Cortical neurons, established from E14.5 mouse embryos and cultured until 3 DIV, immunostained with a TBR1 Ab (green) or with a CTIP2 Ab (green), and with the TuJ1 Ab (red), detecting β3-tubulin; blue: DAPI to visualize nuclei. The histogram on the right represents the percentage of TuJ1+ cells positive for TBR1 (97%) and CTIP2 (64%). Total number of neurons counted: 986 (18 fields) for TBR1 and 912 (20 fields) for CTIP2 (data from 3 biological replicates). (B) left: immunostaining with GAD65, a GABAergic neuron marker, of UMNs harvested at E14.5 and cultured for 14 DIV; no GAD65 signal is detected. Right: cultures established from E17.5 mouse cortices are positive for GAD65 after the same time in vitro. (C) EdU staining of E14.5 UMN cultures after 2 DIV (on the left) and 7 DIV, to quantify proliferating cells. While some EdU-positive cells are present at 2 DIV (arrows), no signal is detected after 7 DIV, indicating mitotic quiescence. (D) Representative images of UMN cultures at 3 DIV immunostained for GFAP (astrocytes), O4 (oligodendrocyte progenitors), or IBA1 (microglia) (green). No glial cells were detected in our cultures. (E-G) Positive controls of glial markers. Immunofluorescence of cerebellar or mesencephalic sections as positive controls of the antibodies used to analyze cell cultures. Sagittal sections of P8 mouse cerebellum show GFAP-positive signals (arrow) in Bergmann glia (E) and O4-positive cells (arrow) in the white matter (F). A coronal section of E18.5 mouse mesencephalon shows IBA1-positive microglial cells (arrow) (G). igl: internal granular layer; egl: external granular layer; wm: white matter. Size bar: 25 μm for panels A-D and 50 μm for panels E-G.

**
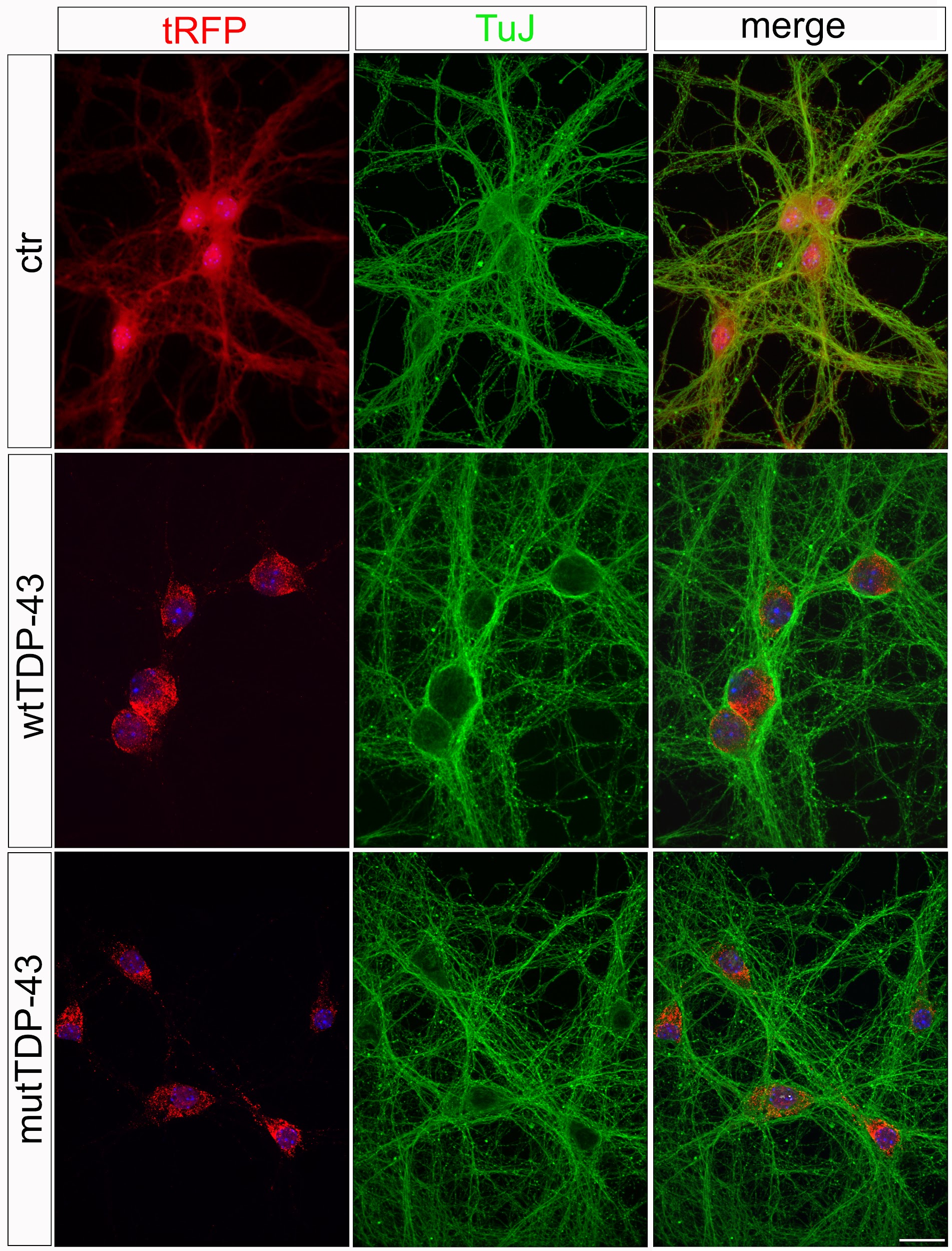
**

**Supplemental figure 2. wtTDP-43 and mutTDP-43 aggregates in all transduced cortical neurons.**

Cortical neuron cultures (ctr, wtTDP-43 and mutTDP-43), established from E14.5 mouse embryos are maintained until 12 DIV. Panels on the left show tRFP fluorescence (red) and nuclear DAPI staining (blue); central panels show TuJ1 immunostaining (neuron-specific β3-tubulin, green). All transduced neurons exhibit cytoplasmic aggregates, are viable, and project a rich network of neurites. Size bar: 20 μm.


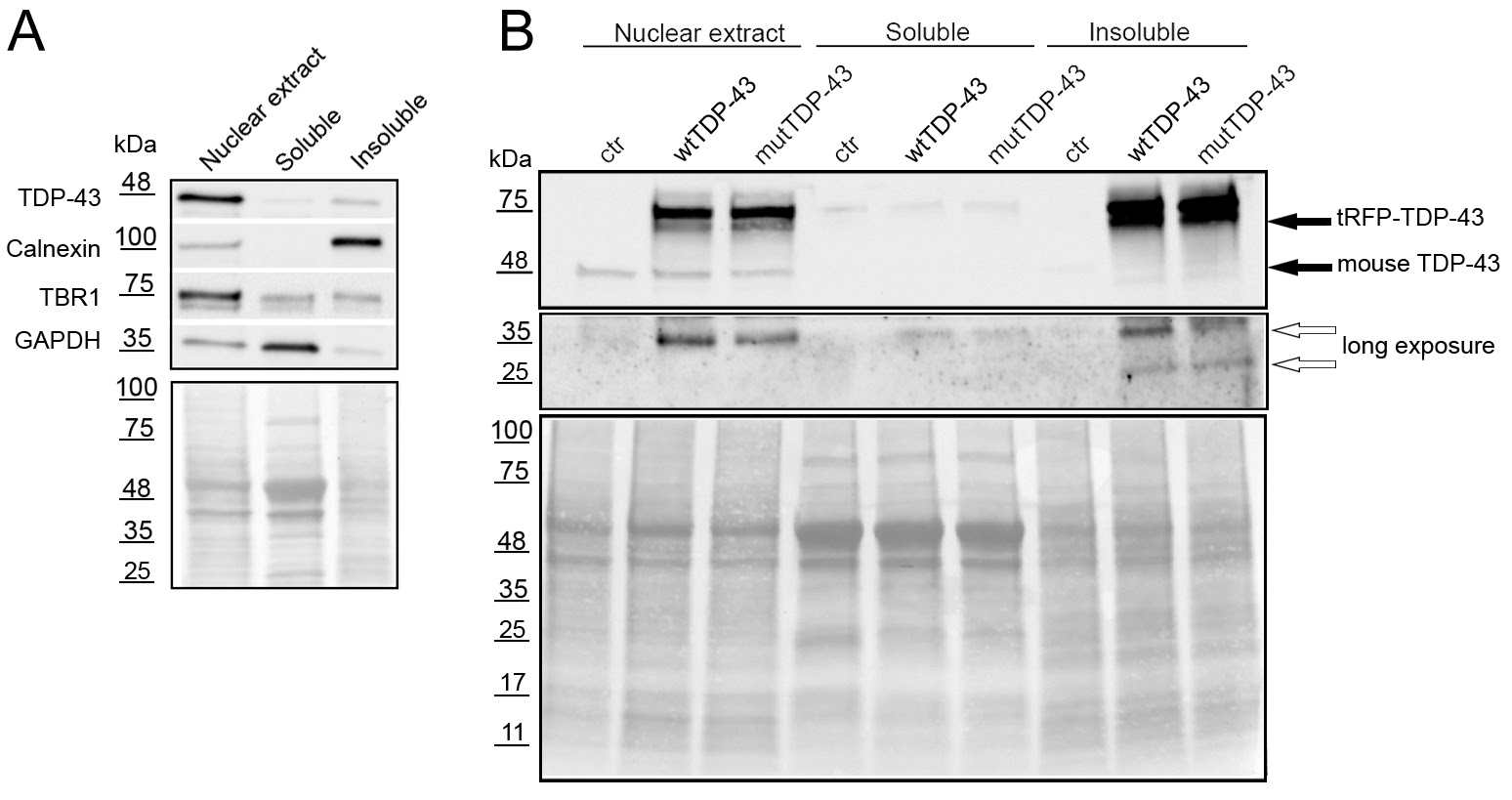


C


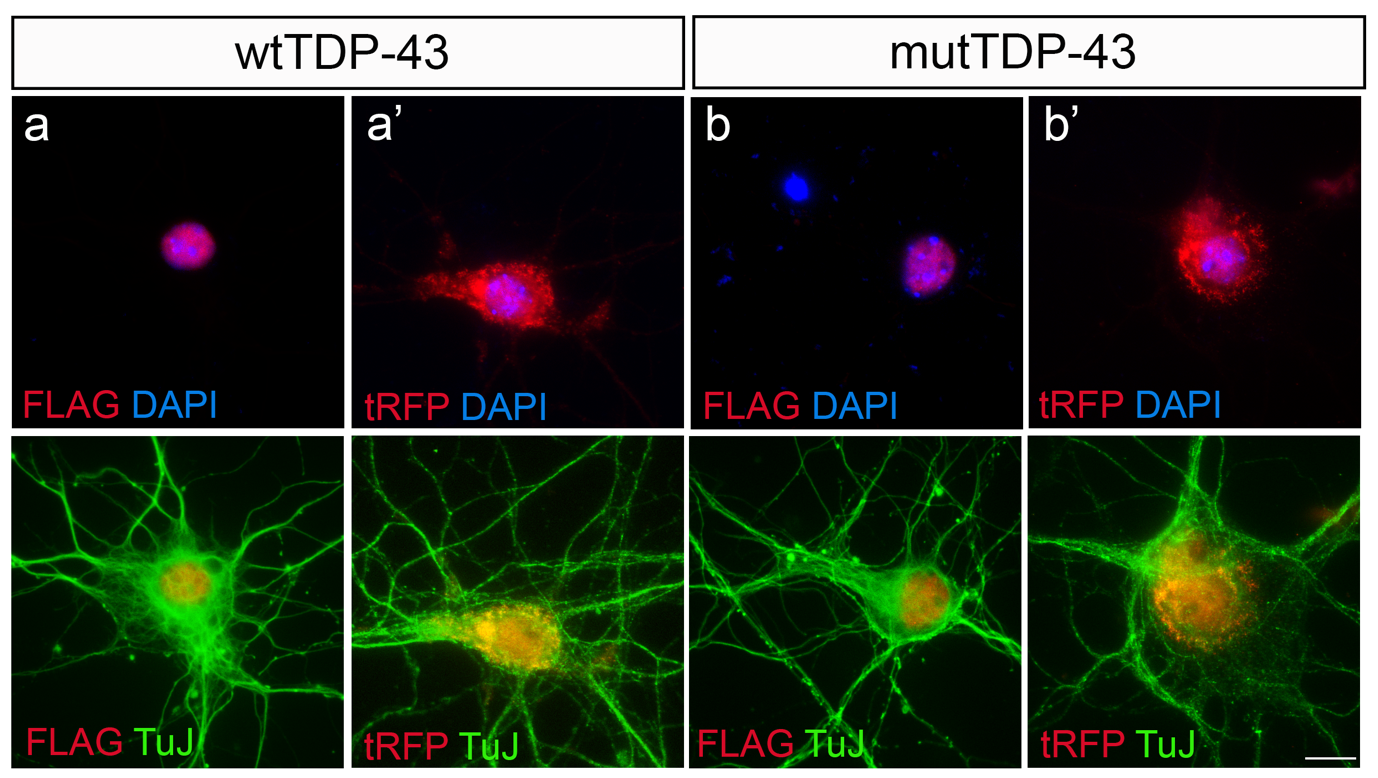


**Supplemental figure 3. Subcellular fractionation of lysates from wtTDP-43 and mutTDP-43 .**

(A) Western blot of the subcellular fractions of proteins extracted from non-transduced cortical neurons at 14 DIV. Endogenous TDP-43 is mainly enriched in the nuclear fraction. Calnexin, a transmembrane protein of the endoplasmic reticulum, and the cytosolic protein GAPDH are enriched in the insoluble cytoplasmic fraction and in the soluble cytoplasmic fraction, respectively. TBR1, a transcription factor highly expressed in cortical neurons, is enriched in nuclear fractions. Ponceau staining is used as a loading control.

(B) Western blot analysis of subcellular fractionation performed on neurons at 14 DIV transduced with ctr, wtTDP-43 and mutTDP-43. Exogenous TDP-43 (75kDa) is particularly abundant in the cytoplasmic insoluble fraction (upper solid arrow). Upon long exposure we observed the presence of additional TDP-43 fragments (empty arrows). Note the presence of 25kDa band only in the insoluble fraction of the TDP-43 overexpressing neurons. C) Cortical neuron cultures (12 DIV) transduced with wtTDP-43 or mutTDP-43 tagged with FLAG (a,b) or tRFP (a’,b’). tRFP fluorescence (red) reveals TDP-43 aggregates in the cytoplasm (a’,b’), while the FLAG-tagged TDP-43 (red in a,b) shows a mostly nuclear localization, indicating that the cytoplasmic localization is accelerated by the tRFP tag and not dependent on overexpression. In the lower gallery the same neurons are also labeled with TuJ antibody (green), indicating that they are viable and healthy. Nuclei are counterstained with DAPI (blue). Size bar: 10 μm.


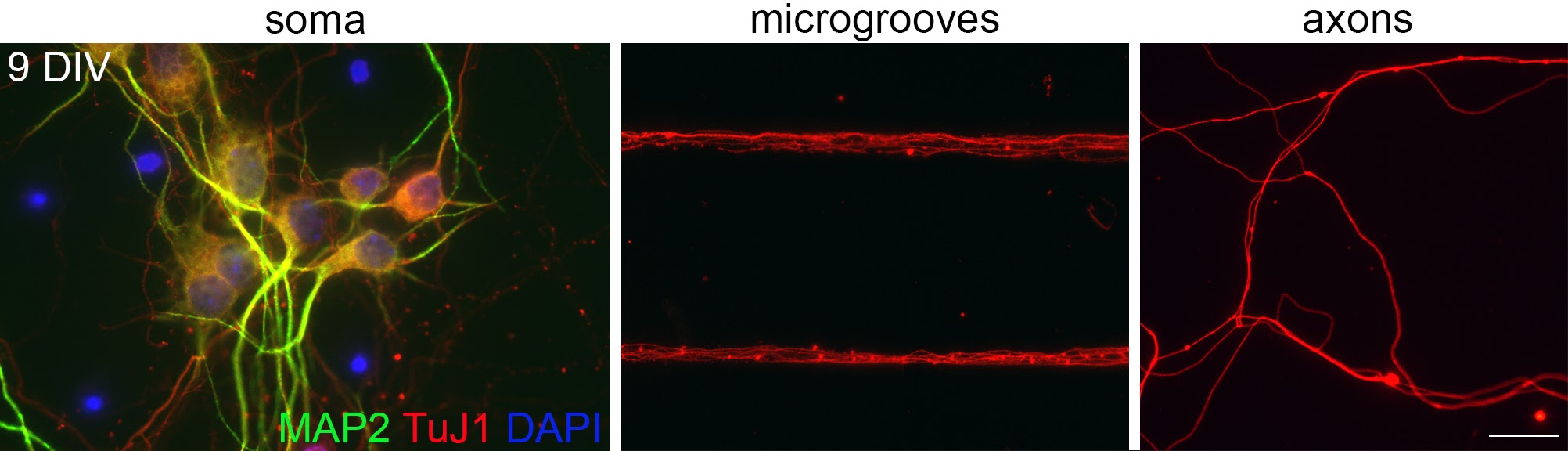


**Supplemental figure 4. Physical separation of whole-cell and axonal compartments in microfluidic chambers.** Immunofluorescence of UMN cultures, grown in microfluidic chambers until 9 DIV, with antibodies for TuJ1 (red), a marker of dendrites and axons alike, MAP2 (green), a dendrite-specific marker, and DAPI, a nuclear marker. Microfluidic chambers allow the physical separation of axons (MAP2-negative) from dendrites (MAP2-positive) and cell bodies. Size bar: 20 μm.


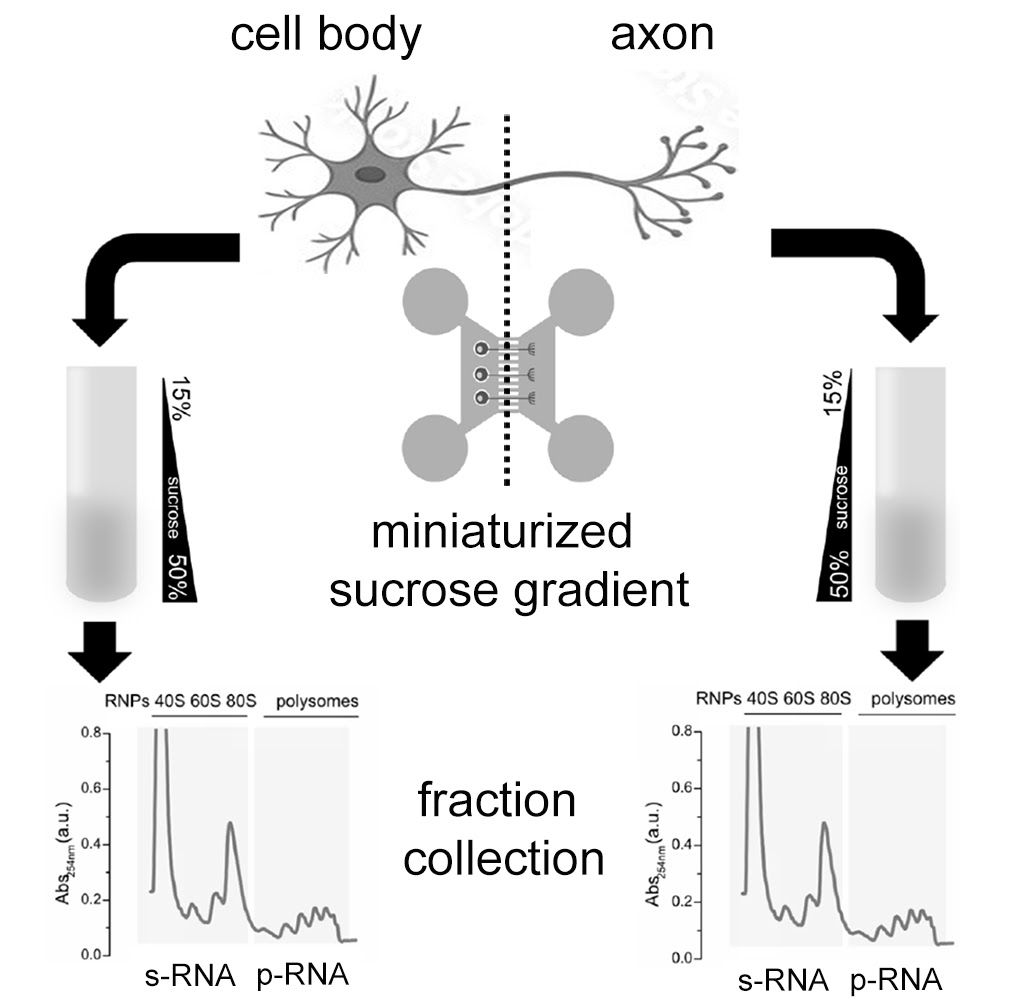


**Supplemental figure 5. Representative scheme of polysome-engaged and sub-polysomal mRNA isolation for sequencing.** Neurons transduced with ctr, wtTDP-43 and mutTDP-43 lentiviral particles were grown in microfluidic chambers until 9DIV. Axonal and whole-cell compartments were lysed and loaded onto a miniaturized sucrose gradient. Then mRNAs from polysomal fractions (p-RNAs, containing polysome-engaged mRNAs) and sub-polysomal fractions (s-RNAs, containing mRNAs not associated with polysomes) were isolated and sequenced.


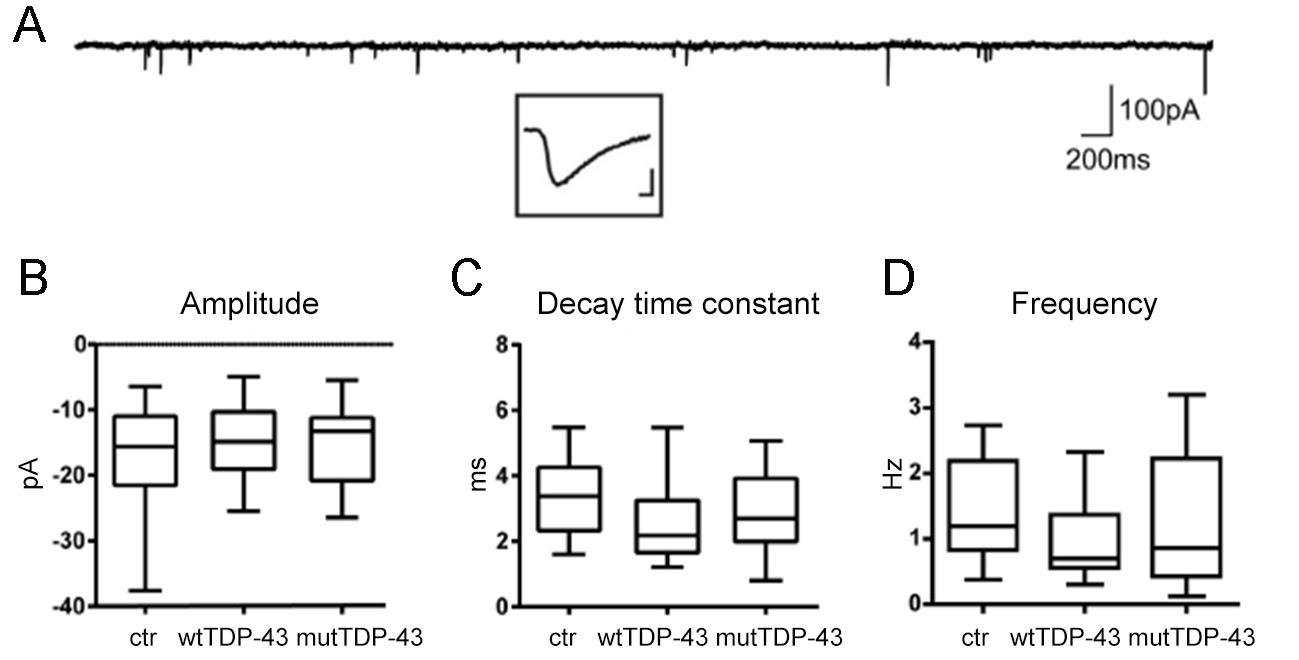


**Supplemental figure 6.** **wtTDP-43 and mutTDP-43 neurons show no significant differences in mEPSC amplitude, decay time constant, and frequency.**

(A) Example of spontaneous mEPSCs recorded in voltage clamp mode (V_h_: -70mV) in a cultured cortical cell. The inset shows an enlarged view of an individual mEPSC (scale bar: 100 pA, 1 ms). (B-D). Summary box plots of peak amplitude, decay time constant, and frequency of mEPSCs recorded in ctr, wtTDP-43, and mutTDP-43 cultures (box plots represent medians, 25 and 75 percentiles, and min-max values, from 16 ctr neurons, 19 wtTDP-43 neurons and 15 mutTDP-43 neurons, 3 experiments for each condition, Mann Whitney U-Test, ns=p≥0.05).

| **Supplemental table 1 – Downregulated axonal mRNAs shared between wtTDP-43 and mutTDP-43 populations** | | | | | |
| --- | --- | --- | --- | --- | --- |
| **Gene ontology** | **gene** | **wtTDP-43 log2FC** | **wtTDP-43 pvalue** | **mutTDP-43 log2FC** | **mutTDP-43 pvalue** |
| **mRNA translation/protein synthesis** | Celf1 | -5,51720 | 0,00768 | -4,39563 | 0,03242 |
|  | Cnot7 | -8,83478 | 0,03222 | -4,19056 | 0,03721 |
|  | Eef1b2 | -4,25090 | 0,04699 | -9,90550 | 0,03620 |
|  | Eif4g1 | -8,56527 | 0,04170 | -11,24886 | 0,00609 |
|  | Eif4g2 | -3,75414 | 0,04867 | -10,60810 | 0,01560 |
|  | Eif4g3 | -9,73778 | 0,01275 | -9,73778 | 0,04335 |
|  | Eif5a | -10,13464 | 0,00780 | -4,42757 | 0,03149 |
|  | Fus | -10,06343 | 0,00889 | -10,06343 | 0,03185 |
|  | Fxr1 | -10,26857 | 0,00659 | -10,26857 | 0,02478 |
|  | Gapdh | -4,18785 | 0,04718 | -9,84240 | 0,03590 |
|  | Gm6576 | -9,69124 | 0,01201 | -9,69124 | 0,04185 |
|  | Gm8210 | -10,72888 | 0,00350 | -10,72888 | 0,01435 |
|  | Hspa8 | -4,93743 | 0,02142 | -10,58520 | 0,01800 |
|  | Ilf3 | -8,96979 | 0,02819 | -9,62581 | 0,04644 |
|  | Myc | -10,26382 | 0,00707 | -10,26380 | 0,02610 |
|  | Nop56 | -9,86366 | 0,01070 | -9,86366 | 0,03748 |
|  | Npm1 | -3,86743 | 0,02925 | -11,82221 | 0,00353 |
|  | Pabpc1 | -9,65693 | 0,01261 | -9,65693 | 0,04352 |
|  | Pcbp2 | -10,29837 | 0,00606 | -10,29837 | 0,02323 |
|  | Ptbp1 | -9,76253 | 0,01223 | -9,76253 | 0,04211 |
|  | Pum2 | -6,29466 | 0,00229 | -11,94253 | 0,00219 |
|  | Rbm4 | -9,62275 | 0,01256 | -9,62275 | 0,04368 |
|  | Rbm4b | -9,54182 | 0,01372 | -9,54182 | 0,04703 |
|  | Rock2 | -9,61718 | 0,01449 | -9,61718 | 0,04820 |
|  | Rpl11 | -9,79642 | 0,01185 | -9,79642 | 0,04072 |
|  | Rpl14 | -3,22454 | 0,04911 | -3,63098 | 0,04067 |
|  | Rpl22 | -4,00094 | 0,03187 | -10,17489 | 0,02737 |
|  | Rpl3 | -3,50682 | 0,04717 | -11,46480 | 0,00580 |
|  | Rpl30 | -10,34846 | 0,00560 | -10,34846 | 0,02176 |
|  | Rpl35a | -10,25323 | 0,00597 | -10,25323 | 0,02320 |
|  | Rpl5 | -3,82593 | 0,03167 | -11,46280 | 0,00510 |
|  | Rpl6 | -10,68039 | 0,00323 | -10,86022 | 0,01263 |
|  | Rpl7 | -10,04731 | 0,00844 | -10,04731 | 0,03075 |
|  | Rpl9 | -10,24448 | 0,00678 | -10,24450 | 0,02540 |
|  | Rplp2 | -9,39192 | 0,01827 | -10,21459 | 0,02742 |
|  | Rps12 | -3,00647 | 0,04913 | -3,60482 | 0,03179 |
|  | Rps13 | -8,50554 | 0,03677 | -10,92460 | 0,01080 |
|  | Rps15a | -11,32712 | 0,00123 | -4,40490 | 0,03290 |
|  | Rps2-ps6 | -9,35582 | 0,01166 | -9,35582 | 0,04370 |
|  | Rps24 | -10,19377 | 0,00717 | -10,19377 | 0,02666 |
|  | Rps7 | -2,96620 | 0,04499 | -12,29037 | 0,00057 |
|  | Rps8 | -3,87188 | 0,04249 | -10,12852 | 0,02724 |
|  | Rps9 | -8,75618 | 0,03489 | -11,63844 | 0,00314 |
|  | Snu13 | -9,23472 | 0,02170 | -10,09159 | 0,02954 |
|  | Srp54b | -9,71318 | 0,01273 | -9,71318 | 0,04338 |
|  | Xpo1 | -3,63965 | 0,04745 | -10,85008 | 0,01183 |
|  | Zfp706 | -8,52080 | 0,04378 | -9,77539 | 0,03643 |
|  |  |  |  |  |  |
| **Gene ontology** | **gene** | **wtTDP-43 log2FC** | **wtTDP-43 pvalue** | **mutTDP-43 log2FC** | **mutTDP-43 pvalue** |
| **Oxidative stress** | Becn1 | -10,00881 | 0,00902 | -10,00881 | 0,03244 |
|  | Clcn3 | -10,13959 | 0,00723 | -10,13959 | 0,02711 |
|  | Hsph1 | -9,52498 | 0,01585 | -9,62136 | 0,04815 |
|  | Map3k7 | -8,52440 | 0,04114 | -10,67157 | 0,01550 |
|  | Mapk9 | -9,48135 | 0,01668 | -9,80488 | 0,04029 |
|  | Nfe2l1 | -4,68459 | 0,02649 | -10,33241 | 0,02160 |
|  | Prdx2 | -9,25213 | 0,02131 | -10,06556 | 0,02999 |
|  | Rock2 | -9,61718 | 0,01449 | -9,61718 | 0,04820 |
|  |  |  |  |  |  |
| **Gene ontology** | **gene** | **wtTDP-43 log2FC** | **wtTDP-43 pvalue** | **mutTDP-43 log2FC** | **mutTDP-43 pvalue** |
| **Synaptic function** | Abi1 | -4,74904 | 0,02480 | -10,40361 | 0,02023 |
|  | Abi2 | -8,98422 | 0,02591 | -9,67504 | 0,04097 |
|  | Abr | -4,79402 | 0,02240 | -10,44859 | 0,01835 |
|  | Actr3 | -9,85165 | 0,01110 | -9,85165 | 0,03856 |
|  | Adgrl1 | -8,91056 | 0,02988 | -11,47194 | 0,00392 |
|  | Adgrl3 | -9,38601 | 0,01292 | -9,38601 | 0,04649 |
|  | Afdn | -9,21828 | 0,02212 | -10,17164 | 0,02557 |
|  | Agrn | -9,55412 | 0,01505 | -9,55412 | 0,04995 |
|  | Agtpbp1 | -9,53461 | 0,01391 | -9,53461 | 0,04753 |
|  | Akap7 | -9,52666 | 0,01488 | -9,52666 | 0,04979 |
|  | Ap1ar | -9,61530 | 0,01261 | -9,61530 | 0,04385 |
|  | Apbb1 | -10,05419 | 0,00758 | -10,05419 | 0,02850 |
|  | Arhgap21 | -5,11026 | 0,01197 | -3,98180 | 0,04966 |
|  | Arhgef9 | -9,58812 | 0,01495 | -9,58812 | 0,04964 |
|  | Atp2b2 | -10,06425 | 0,00844 | -10,06425 | 0,03063 |
|  | Bsg | -9,95800 | 0,00983 | -9,76085 | 0,04243 |
|  | Cadm1 | -8,61658 | 0,03771 | -10,21661 | 0,02112 |
|  | Camk2d | -9,84210 | 0,01118 | -9,84210 | 0,03905 |
|  | Cask | -8,81330 | 0,03264 | -9,66202 | 0,04183 |
|  | Clcn3 | -10,13959 | 0,00723 | -10,13959 | 0,02711 |
|  | Clta | -9,57558 | 0,01493 | -9,57558 | 0,04950 |
|  | Copa | -9,12430 | 0,02270 | -10,05412 | 0,03204 |
|  | Copg2 | -11,03324 | 0,00281 | -11,03324 | 0,01146 |
|  | Cux1 | -8,45220 | 0,04653 | -10,10680 | 0,02680 |
|  | Dclk1 | -9,68729 | 0,01305 | -9,68730 | 0,04430 |
|  | Dlg4 | -10,13707 | 0,00487 | -10,13707 | 0,02068 |
|  | Dlgap1 | -9,92957 | 0,00764 | -9,92957 | 0,02940 |
|  | Dst | -10,51926 | 0,00403 | -10,51926 | 0,01662 |
|  | Epb41l2 | -8,56990 | 0,04156 | -9,68223 | 0,04555 |
|  | Erbin | -9,74838 | 0,01255 | -9,74838 | 0,04290 |
|  | Flna | -10,96409 | 0,00235 | -10,96409 | 0,01021 |
|  | Fus | -10,06343 | 0,00889 | -10,06343 | 0,03185 |
|  | Fxr1 | -10,26857 | 0,00659 | -10,26857 | 0,02478 |
|  | Gapdh | -4,18785 | 0,04718 | -9,84242 | 0,03590 |
|  | Gapvd1 | -4,29656 | 0,04392 | -9,94438 | 0,03419 |
|  | Gdi1 | -8,84440 | 0,03125 | -10,53450 | 0,01625 |
|  | Gls | -8,52445 | 0,04332 | -10,11239 | 0,02890 |
|  | Gnas | -10,02253 | 0,00772 | -10,02253 | 0,02906 |
|  | Gpm6b | -9,68081 | 0,01287 | -5,48103 | 0,00467 |
|  | Hnrnpa2b1 | -9,57877 | 0,01513 | -10,87230 | 0,01170 |
|  | Hspa8 | -4,93743 | 0,02142 | -10,58525 | 0,01800 |
|  | Kif3c | -8,32199 | 0,04903 | -9,76387 | 0,03810 |
|  | Macf1 | -3,73722 | 0,04784 | -10,36876 | 0,01871 |
|  | Malat1 | -10,75198 | 0,00193 | -10,75198 | 0,00935 |
|  | Map2 | -9,62409 | 0,01211 | -9,62409 | 0,04260 |
|  | Map3k7 | -8,52440 | 0,04114 | -10,67157 | 0,01550 |
|  | Mapk9 | -9,48135 | 0,01668 | -9,80488 | 0,04029 |
|  | Mia2 | -9,82497 | 0,01155 | -9,82497 | 0,03996 |
|  | Ncoa2 | -4,19161 | 0,04761 | -9,83943 | 0,03651 |
|  | Ndrg4 | -8,64301 | 0,03491 | -10,65132 | 0,01562 |
|  | Nfia | -10,32364 | 0,00402 | -10,32364 | 0,01736 |
|  | Nrxn2 | -10,16460 | 0,00655 | -10,16460 | 0,02520 |
|  | Pak3 | -10,18450 | 0,00459 | -10,18450 | 0,01960 |
|  | Pebp1 | -9,68111 | 0,01352 | -9,68111 | 0,04551 |
|  | Plekha5 | -9,85946 | 0,01115 | -9,85946 | 0,03866 |
|  | Psma3 | -8,90291 | 0,03029 | -9,90581 | 0,03475 |
|  | Ptk2 | -9,08819 | 0,02456 | -9,76728 | 0,03864 |
|  | Rapgef1 | -9,64328 | 0,01195 | -9,64328 | 0,04207 |
|  | Rims1 | -9,27216 | 0,02093 | -10,04670 | 0,02900 |
|  | Rnf10 | -5,20284 | 0,00952 | -10,85072 | 0,00825 |
|  | Rock2 | -9,61718 | 0,01449 | -9,61718 | 0,04820 |
|  | Scn1a | -9,58428 | 0,01463 | -9,58428 | 0,04876 |
|  | Scn8a | -10,52373 | 0,00426 | -10,52373 | 0,01726 |
|  | Sgip1 | -9,52660 | 0,01446 | -9,52660 | 0,04883 |
|  | Sh3kbp1 | -9,20360 | 0,02172 | -10,02933 | 0,02921 |
|  | Stxbp1 | -3,76090 | 0,04969 | -10,39240 | 0,01980 |
|  | Syne1 | -8,35567 | 0,04516 | -9,78040 | 0,04160 |
|  | Synj1 | -9,99539 | 0,00854 | -9,99540 | 0,03130 |
|  | Syt7 | -9,93327 | 0,00840 | -9,93330 | 0,03130 |
|  | Tln2 | -9,88031 | 0,01084 | -9,88030 | 0,03790 |
|  | Tmcc1 | -9,33689 | 0,01804 | -9,74650 | 0,04300 |
|  | Tmod2 | -9,07677 | 0,02550 | -10,18170 | 0,02250 |
|  | Tnik | -3,78642 | 0,04579 | -10,42320 | 0,01790 |
|  | Ube2i | -9,78310 | 0,01154 | -9,78310 | 0,04010 |
|  | Usp48 | -4,24910 | 0,04565 | -9,90370 | 0,03510 |
|  | Ywhaq | -10,15046 | 0,00771 | -10,15046 | 0,02829 |
|  | Zfp365 | -9,82286 | 0,01148 | -9,82290 | 0,03960 |

| **Sup. Table 2** |  |  |  |  |
| --- | --- | --- | --- | --- |
| **Primary Antibodies for Immunofluorescence** | | | | |
| **Description** | **Brand** | **Cat. #** | **Host/ isotype** | **Dilution** |
| Map2 | Millipore | MAB3418 | Mouse/IgG | 1:1000 |
| TDP-43 | Proteintech | 10782-2-AP | Rabbit/IgG | 1:500 |
| βIII-Tubulin | Covance | MMS-435P | Mouse/IgG2a | 1:1000 |
| βIII-Tubulin | BioLegend | 802001 | Rabbit/IgG | 1:1000 |
| G3BP1 | Proteintech | 13057-2-AP | Rabbit/IgG | 1:300 |
| HuC/D | Thermo Fisher | A-21271 | Mouse/IgG2b | 1:2000 |
| tRFP | Evrogen | AB233 | Rabbit/IgG | 1:1000 |
| O4 | Sigma | O7139 | Mouse/IgM | 1:100 |
| CTIP2 | Abcam | ab18465 | Rat/IgG | 1:250 |
| GFAP | DakoCytomation | Z0334 | Rabbit/IgG | 1:500 |
| IBA1 | Wako | 1919741 | Rabbit/IgG | 1:500 |
| TBR1 | Abcam | ab31940 | Rabbit/IgG | 1:250 |
| GAD65 | GeneTex | GTX100281 | Rabbit/IgG | 1:100 |
| RPS6 | Cell signaling | mAb#2217 | Rabbit/IgG | 1:500 |
| RPL26 | BethylLab | A300-686AT | Rabbit/IgG | 1:500 |
| Caspase3 | BD PharMingen | 559565 | Rabbit/IgG | 1:200 |
| Puromycin | Millipore | MABE343 | Mouse/IgG | 1:100 |
| **Primary Antibodies for Western Blotting** | | | | |
| TDP-43 | Proteintech | 10782-2-AP | Rabbit | 1:1000 |
| Calnexin | Sigma | C4731 | Rabbit | 1:5000 |
| tRFP | Evrogen | AB233 | Rabbit | 1:5000 |
| GAPDH | Santa Cruz | sc-25778 | Rabbit | 1:2500 |
| Histone-H3 | Abcam | ab1791 | Rabbit | 1:5000 |
| **Secondary Antibodies for immunofluorescence** | | | | |
| **Description** | **Brand** | **Cat. #** | | **Dilution** |
| Alexa FluorTM 546 Goat anti-mouse (IgG) | Invitrogen | A11003 | | 1:1000 |
| Alexa FluorTM 546 Goat anti-mouse (IgG2a) | Invitrogen | A21133 | | 1:1000 |
| Alexa FluorTM 488 Goat anti-rabbit (IgG) | Invitrogen | A11034 | | 1:1000 |
| Alexa FluorTM 488 Goat anti-mouse (IgG) | Invitrogen | A11001 | | 1:1000 |
| Alexa FluorTM 488 Goat anti-mouse (IgG2a) | Invitrogen | A21131 | | 1:1000 |
| Alexa FluorTM 647 Goat anti-mouse (IgG) | Invitrogen | A32728 | | 1:1000 |
| Alexa FluorTM 647 Goat anti-rabbit (IgG) | Invitrogen | A21244 | | 1:1000 |
| **Secondary Antibodies for Western Blotting** | | | | |
| Goat anti-mouse IgG (H+L)-HRP Conjugate | Biorad | 172-1011 | | 1:10000 |
| Goat anti-rabbit IgG (H+L)-HRP Conjugate | Biorad | 170-6515 | | 1:10000 |
